# Comparison between the Ability of Glasgow Coma Scale and Full Outline of Unresponsiveness Score to Predict the Mortality and Discharge Rate of Pediatric Intensive Care Unit Patients 

**Published:** 2014-09-12

**Authors:** Ali Khajeh, Afshin Fayyazi, Ghasem Miri-Aliabad, Hasan Askari, Noormohammad Noori, Behrouz Khajeh

**Affiliations:** Children and Adolescence Health Research Center, Zahedan University of Medical Sciences, Zahedan, Iran

**Keywords:** Glasgow Coma Consciousness, FOUR Score, Mortality, PICU, GCS

## Abstract

***Objective:*** Prediction of survival and mortality rates in costly environments such as the intensive care unit (ICU) is of great importance for the assessment of new treatments, resource consumption control, and improvement of quality control. This study aimed to determine the ability to predict mortality and discharge rate of patients using the FOUR score in the pediatric ICU (PICU) of Ali Ibn Abitalib Hospital, Zahedan and compare the results with those of Glasgow Coma Scale (GCS).

***Methods:*** This prospective study was conducted on 200 patients admitted to the PICU. Convenience purposive sampling was used. Research data was collected using the Full Outline of Unresponsiveness (FOUR) score and GCS using questionnaires. Obtained data was analyzed with SPSS 16 using descriptive statistics and correlation analyses.

***Findings:*** Of the 200 children admitted to the PICU, 71.5% and 28.5% were discharged and died, respectively. The inter-rater reliability for the FOUR score was good to excellent (weighted κ: eye, 0.72; respiration, 0.82; brainstem, 0.74; motor, 0.78), In terms of mortality and discharge prediction, logistic regression analyses (FOUR score = OR: 0.13; 95% CI: 0.06–0.29; *P*<0.001; GCS=OR: 2.49; 95% CI: 1.44–4.32; *P*<0.001) showed that the FOUR score is a good predictor for in-hospital mortality.

***Conclusion:*** Results indicated that the FOUR score is more capable than GCS in predicting the mortality and discharge of patients admitted to the PICU.

## Introduction

Considering the high mortality in intensive care units (ICUs) in hospitals compared with other units as well as high costs of inpatient treatment in these units, mortality prediction has long been a concern^[^^1^^]^. Several tools are designed for mortality prediction in ICUs^[^^2^^]^. One of the most widely used tools for examining patients’ consciousness level and disease outcome prediction is Glasgow Coma Scale (GCS)^[^^3^^,^^4^^]^. This scale was first developed in 1974 to evaluate the consciousness level of head injury patients^[^^5^^]^, and then was widely used for evaluating the consciousness level of other patients admitted to ICU^[^^6^^]^. Several studies have indicated that GCS provides the guideline for primary care and disease outcome prediction (mortality and morbidity)^[^^7^^-^^9^^]^. Because of the failure of GCS in examining the verbal responses of intubated patients and evaluating brainstem reflexes, several other scales have become popular for assessment of intubated patients’ consciousness level and disease outcome prediction during the past decade. However, none of the other scales have been used widely^[^^3^^,^^10^^-^^12^^]^. During recent years, many efforts to improve CGS have been made so that it can be used more easily. One of these tools is the Full Outline of Unresponsiveness (FOUR) score that was designed by Wijdicks et al in 2005^[^^12^^]^. This scale includes four considerable components: eye responses, motor responses, brainstem reflexes, and breathing pattern. Each component receives a score between 0 and 4 (the lowest and highest scores are 0 and 4, respectively)^[^^13^^]^. Several studies have investigated the validity of the FOUR score and suggest that it is a good alternative for GCS in disease outcome prediction^[^^14^^-^^17^^]^.

 The results of the research by Cohen on 60 children admitted to an ICU in California (2009) indicated that the FOUR score is a powerful tool in disease outcome prediction for pediatric patients admitted to ICU and the inter-rater reliability for the FOUR score was excellent^[^^14^^]^.

 Because a large percentage of patients admitted to ICU are comatose^[^^18^^]^, their examination is an important part of work in the ICU, and the most widely used tool for assessing patients’ level of consciousness and predicting disease outcome is GCS. Because of the weaknesses of GCS and its failure in assessing verbal responses in intubated patients, the brainstem reflexes and also the strengths of the FOUR score in brainstem reflex assessment, we decided to compare the ability of GCS and FOUR score in predicting the mortality and discharge of patients admitted to pediatric ICU (PICU).

## Subjects and Methods

This prospective study was conducted in the PICU of Ali Ibn Abitalib Hospital, Zahedan. The Children and Adolescents Health Research Center of Zahedan University of Medical Sciences approved the study. Convenience purposive sampling was used. Written consent was obtained from family members of patients, and they were assured that patient’s personal information would be safe and would be used only for research and they could withdraw from the study any time. Sample size was calculated at 200 according to the sample size formula. Inclusion criteria were all children with neurological or neurosurgery disorders admitted to PICU of the Hospital. Exclusion criteria included patients receiving sedating drugs and neuromuscular blockers including midazolam, fentanyl, sufentanil, morphine, pancuronium bromide, atracurium, nesdonal, and propofol, or had recognized vision, hearing, speech, or limb paralysis problems. In addition, patients under the age of two years and above 12 years (because of an inability to communicate verbally ill patients less than 2 years and because of lack of PICU admission in patients over 12 years) were excluded. Data collection lasted from February to November 2012. Data was collected using the FOUR score and GCS using questionnaires. The patients’ level of consciousness was routinely controlled by nurses using GCS after entering the PICU and recorded in a special flowchart for consciousness level measurement. To measure the patients’ level of consciousness using the FOUR score, it was primarily translated to Persian and then back-translated to English, and the accordance of English versions were examined by an individual fluent in both languages. Content validity index (CVI) was used for measuring the validity of the data collection tool. Ten faculty members of the department of neurology and neurosurgery were provided with the tool, and their comments and corrections were applied. The new coma scale (FOUR score) was taught to nurses participating in this study by the specialty pediatric neurology during three 30–45 min sessions on each item (Table 1)^[^^16^^]^. And each participant was given an instruction booklet regarding the FOUR score. And again after a week of personal training, clinical training in the PICU was given by a pediatric neurologist. At the end of the course, each of the nurses participating in the study were allowed to practice on 2–3 patients and all the problems were resolved in relation to working with this scale. Sixteen nurses participated in the study. All of these 16 nurses had bachelor’s degree in nursing. 

**Table 1:** Full Outline of Unresponsiveness (FOUR) score^[^^16^^]^

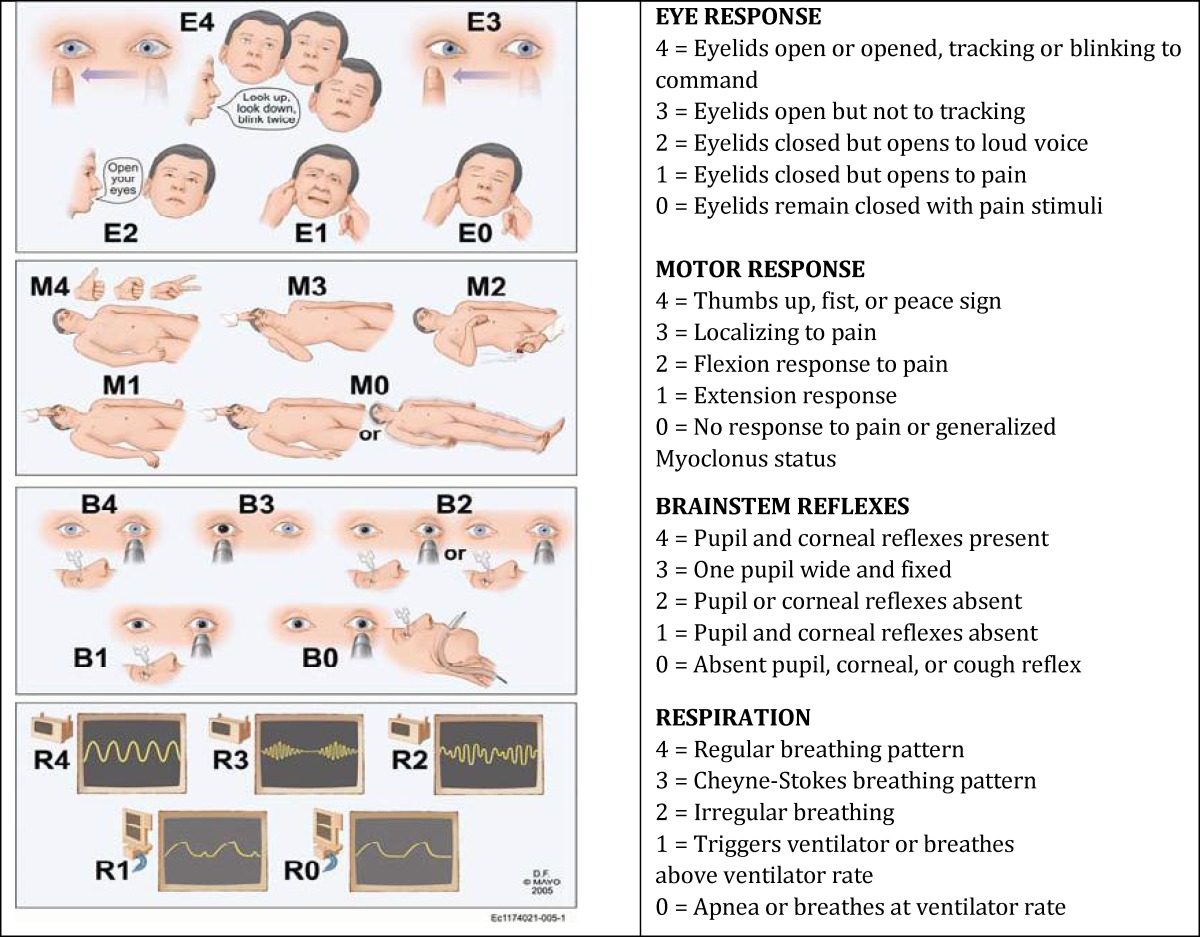

Nurses had different working shifts (morning, evening, and night) and different working experience including recruiting, contract, and formal nurses, and the average work experience was 8.31±7.14 years. To assess inter-rater reliability of the FOUR score, each patient was rated on the FOUR score by two differently trained nurses. The raters performed their examination on arrival of the patient to the PICU without knowledge of the other rater’s scores. To study the predictive ability of mortality and discharge rate of both scales, scores of the FOUR score were compared to those of GCS, which were routinely controlled by nurses and recorded in the special flowchart for measuring GCS scores. For patients who had undergone intubation, the lowest GCS verbal score was used both for scoring and for data analysis. Ultimately, both tools were compared regarding their predictability of patient mortality or discharge. Afterwards, obtained data was analyzed using SPSS 16.

## Findings

Of the 200 patients that participated in this research, 55% (n=110) were males and 45% (n=90) females. The mean age of patients was 4.4 years. Of the 200 patients, 76% (n=152) had spontaneous respiration and 24% (n=48) were ventilated with a mechanical ventilator. The cause of patients’ admission to ICU was mostly intracranial hemorrhage. The admission diagnoses of patients are listed in Table 2. Of the 200 patients who participated in this study, 143 (71.5%) patients were discharged after recovery and 57 (28.5%) patients died in ICU. According to the results of the independent t test, patients’ age did not affect the outcome (discharge or death) (*P*=0.5). Also, results of the chi square test did not show any differences with regard to the outcome and patients’ sex (*P*=0.5). The inter-rater reliability of the FOUR score was evaluated using the weighted kappa (κ_w_) coefficient.

**Table 2:** Admission diagnosis of patients

**Diagnosis**	**Number (Percent)**
**Intracranial hemorrhage**	36 (18)
**Intracranial infection**	31 (15.5)
**Hydrocephaly**	29 (14.5)
**Aneurism**	28 (14)
**Seizure**	27 (13.5)
**Brain tumor**	22 (11)
**Other causes**	27 (13.5)

A κ_w_ statistic of ≤0.4 is considered poor, values between 0.4 and 0.6 are considered fair to moderate, those between 0.6 and 0.8 suggest good inter-observer agreement, and values greater than 0.8 suggest excellent agreement. The rater agreement is shown in Table 3. The inter-rater reliability for the FOUR score was good to excellent (weighted κ: eye, 0.72; respiration, 0.82; brainstem, 0.74; motor, 0.78).

 The mean score of the FOUR and GCS at the time of ICU admission for all patients was 10.5±4.1 (range: 0–16) and 10.4±3.9 (range: 3–15), respectively. Mean of the FOUR score at the time of admission was 12.5±2.1 and 5.1±2.8 for discharged and dead patients, respectively (cut-off point 8) (Table 4). The differences between the two groups were statistically significant (*P*=0.001). The mean GCS at the time of admission was 11.4±3.5 and 7.9±3.8 for discharged and dead patients, respectively (cut-off point 9; *P*=0.001). Logistic regression analysis was performed to determine the ability of the two scales (GCS and FOUR score) to predict the outcome. Results of this test showed that odds ratios for the FOUR score are somewhat lower than those for the GCS (FOUR score=OR: 0.13; 95%CI: 0.06–0.29; *P*<0.001; GCS=OR: 2.49; 95%CI: 1.44–4.32; *P*<0.001). In previous studies, lower odds ratios have been related to a positive predictive value for a higher chance of a positive outcome with increased total score values^[^^13^^,^^16^^]^.

## Discussion

The purpose of establishing a PICU is to obtain the best results and better outcomes for severely ill children. One of the ways to achieve that goal is to predict the mortality risk of the patients admitted to the PICU to provide them with the best care available^[^^20^^]^. It is necessary to develop models that predict the mortality risk in PICU to monitor the effectiveness of the care carried out^[^^21^^]^. For this purpose, the neurological examination tools or coma examination scales of patients are accepted as effective scales for disease outcome examination^[^^2^^]^. To be an effective tool, a coma scale must be practical for use in a wide variety of settings and by healthcare providers with diverse experience^[^^14^^]^. In this regard, the FOUR score is designed to remedy the deficiencies of GCS to show more neurological details in unconscious patients and predict the final result more accurately and easily^[^^4^^,^^16^^]^. Research results indicated that the inter-rater agreement with the FOUR score was good to excellent (weighted κ: eye, 0.72; respiration, 0.82; brainstem, 0.74; motor, 0.78). These results are consistent with those by Wolf et al^[^^11^^]^ and Wijdicks et al^[^^16^^]^. The high level of agreement between nurse raters using the FOUR score suggests that the application of the FOUR score and assessment of the level of consciousness is easier and requires minimal facilities, and nurses with differing levels of experience and expertise are more likely to correctly assess the patient and assign the same score using the FOUR score.

**Table 3:** Kappa values, Standard Error and 95% Confidence Intervals for Inter-rater agreement on the Full Outline of Unresponsiveness score

	**Eye**	**Motor**	**Brainstem**	**Respiration**
**Kappa**	0.72	0.78	0.74	0.82
**Standard Error**	0.037	0.035	0.039	0.032
**Confidence Intervals (CI)**	0.67-0.77	0.73-0.84	0.69-0.80	0.77-0.87

**Table 4:** Mean score of FOUR coma sub score in discharged and deceased patients

**FOUR coma sub scale**		**Number**	**Mean**	***P.*** ** value**
**Eye opening**	**Discharged**	143	2.7 (0.97)	0.001
	**Deceased**	57	0.73 (0.76)	
**Motor**	**Discharged**	143	3.2 (0.82)	0.001
	**Deceased**	57	1.6 (1.01)	
**Brainstem**	**Discharged**	143	3.4 (0.7)	0.001
	**Deceased**	57	1.6 (0.88)	
**Respiration**	**Discharged**	143	3.1 (0.74)	0.001
	**Deceased**	57	1.1 (0.88)	
**Total score**	**Discharged**	143	12.5 (2.1)	0.001
	**Deceased**	57	5.1 (2.8)	

FOUR: Full Outline of Unresponsiveness

 Although the GCS has been widely used in hospital settings, because of the failure in examining the verbal responses of intubated patients and evaluating brainstem reflexes, the FOUR score was developed. By these advantages, the FOUR score can show patients’ real state of consciousness. Therefore, it is better at predicting patients’ future state^[^^3^^,^^14^^]^. 

 Our results demonstrate that mortality in PICU patients with the lowest FOUR score is higher than in patients with the lowest GCS. The mortality rate for patients with the lowest FOUR score of 0 (100%) was higher than that for patients with the lowest GCS score of 3 (85.7%). With this finding, the FOUR score would have great value for outcomes prediction than the GCS. These results are consistent with those by Cohen^[^^14^^]^, Wijdicks et al^[^^16^^]^, and Iyer et al^[^^15^^]^. In the research by Büyükcam et al in Turkey, no significant difference was observed between these tools for predicting the mortality of children admitted to the ICU^[^^19^^]^. This difference is probably because of the fact that the participants of the research by Büyükcam et al were only children with a medical diagnosis of stroke, but in the present research a group of children with different medical neurology and neurosurgery diagnoses were investigated.

 This new coma scale, unlike the GCS, does not include a verbal response, and thus is more valuable in PICU that typically has a large number of intubated patients. In our study, 24% of patients were intubated, and GCS was less useful for verbal response.

Research results indicated that cut off point 8 correlated with worse outcome, while the research by Wijdicks et al^[^^16^^]^ indicated that a cut-off point of 9 and that by Akavipat et al^[^^2^^]^ a cut-off point of 10 correlated with worse outcome^,^. This difference may be due to deterioration of the patients’ health participating in the study.

 A limitation of this study was that the population in this study included only patients with neurological problems and the results of this study cannot be extended to all patients admitted to PICU.

## Conclusion

It is important to assess the consciousness level of patients admitted to PICU using an accurate, easy-to-use tool that is better at showing disease outcome. With respect to the results, the FOUR score is more capable than GCS in assessing patients’ level of consciousness and disease outcome predictability.
